# Young women’s autonomy and information needs in the schools-based HPV vaccination programme: a qualitative study

**DOI:** 10.1186/s12889-020-09815-x

**Published:** 2020-11-10

**Authors:** Harriet Fisher, Karen Evans, Jo Ferrie, Julie Yates, Marion Roderick, Suzanne Audrey

**Affiliations:** 1 Population Health Sciences, Bristol Medical School, University of Bristol, Canynge Hall, 39 Whatley Road, Bristol, BS8 2PS UK; 2 Head of School Nursing and Specialist Nursing Services, Sirona Health & Care, Bristol, South Gloucestershire UK; 3 Screening and Immunisations South West, Public Health England, Bristol, UK; 4 Department of Paediatric Immunology & Infectious Diseases, University Hospitals Bristol and Weston NHS Trust, Bristol, UK

**Keywords:** Autonomy, Information needs, Young people, Vaccination, Communication

## Abstract

**Background:**

Until 2019, the English schools-based human papillomavirus (HPV) vaccination programme was offered to young women (but not young men) aged 12 to 13 years to reduce HPV-related morbidity and mortality. The aim of this study is to explore the extent to which young women were able to exercise autonomy within the HPV vaccination programme. We consider the perspectives of young women, parents and professionals and how this was influenced by the content and form of information provided.

**Methods:**

Recruitment was facilitated through a healthcare organisation, schools and community organisations in a local authority in the south-west of England. Researcher observations of HPV vaccination sessions were carried out in three schools. Semi-structured interviews took place with 53 participants (young women, parents of adolescent children, school staff and immunisation nurses) during the 2017/18 and 2018/19 programme years. Interviews were recorded digitally and transcribed verbatim. Thematic analysis was undertaken, assisted by NVivo software.

**Results:**

Young women’s active participation and independence within the HPV vaccination programme was constrained by the setting of vaccination and the primacy of parental consent procedures. The authoritarian school structure influenced the degree to which young women were able to actively participate in decisions about the HPV vaccination programme. Young women exercised some power, either to avoid or receive the vaccine, by intercepting parental consent forms and procedures. Reliance on leaflets to communicate information led to unmet information needs for young women and their families. Communication may be improved by healthcare professional advocacy, accessible formats of information, and delivery of educational sessions.

**Conclusions:**

Strategies to improve communication about the HPV vaccine may increase young people’s autonomy in consent procedures, clarify young people’s rights and responsibilities in relation to their health care services, and result in higher uptake of the HPV vaccination programme.

**Trial registration:**

ISRCTN 49086105; Date of registration: 12 January 2018; Prospectively registered.

**Supplementary Information:**

The online version contains supplementary material available at 10.1186/s12889-020-09815-x.

## Background

The English human papillomavirus (HPV) vaccination programme was delivered to young women aged 12 to 13 years old from 2009, with the programme being expanded to include young men in 2019. The current vaccine protects against infection from HPV types 6, 11, 16, and 18 which cause genital warts and HPV-related cancers affecting both men and women. High coverage was achieved in the English HPV vaccination programme delivered to young women [1].

Recent evidence for population-level effectiveness has highlighted the potential for HPV vaccination programmes to eradicate cervical cancer [2, 3]. However, national data often conceal within-country inequalities in uptake and access. Wide variations in uptake of the English HPV vaccination programme across local authorities are apparent (range: 70.2–95.8% for the first dose in 2018/19) [1]. In the south-west of England, lower uptake by area and amongst some population groups has been shown [4].

Over the past four decades there has been increasing interest in young people’s rights to self-determination or participation. Globally, the United Nations Convention on the Rights of the Child recognises the right for all children and young people to participate in decision-making processes which involve them [5]. In certain areas of healthcare, predominantly sexual and reproductive treatment, there is now a precedent to consider young people’s capacity to self-consent, as parental consent is viewed as counterproductive in ensuring access to preventative treatment [6].

In England the legal framework allows adolescents to be vaccinated without parental consent if they are assessed as Gillick competent (believed to have enough intelligence, competence and understanding to fully appreciate what is involved in their medical treatment). Despite this legal framework, our previous work demonstrated that the requirement for written parental consent was a barrier to uptake of the HPV vaccination for some young women [7]. This has the potential to exacerbate health inequities in relation to the incidence and mortality of cervical cancer [8–10].

This has the potential to exacerbate health inequities in relation the incidence and mortality of cervical cancer. Although national policies do permit self-consent, a systematic review and evidence synthesis found adolescent self-consent can be undermined by local policies, misunderstandings of the legal framework, and a strong preference for written parental consent to protect the reputation of professionals and relationships between schools and parents. Further, maintaining the role of parents as decision-makers for their child’s healthcare was frequently prioritised over enabling young people’s autonomy to consent [11].

To increase uptake of the HPV vaccination programme, new consent procedures were developed and implemented in two local authority areas in the south-west of England [12]. Delivery of the schools-based vaccination programme had previously required written parental consent. The new procedures included seeking consent verbally from parents on the day of the vaccination session and, where this was not achieved, allowing adolescent self-consent for young women assessed as competent by the immunisation team. An evaluation of the impact of the new consent procedures showed uptake was improved in one of the two local authorities [13]. Further, the additional steps within the procedures addressed some inequalities in uptake by overcoming barriers to vaccination for young women whose families were less likely to respond to paper-based methods of consent (Fisher H, et al.: Stages to increase uptake of the schools-based human papillomavirus (HPV) vaccination programme, submitted).

As part of the evaluation, we also undertook qualitative research to gather evidence in relation to the acceptability of the new consent procedures from the perspectives of young women, parents of male and female adolescent children, school staff and immunisation nurses. Similar to the findings from our systematic review [11], we found reluctance to endorse adolescent self-consent was influenced by the age at which the HPV vaccination is administered (12 to 13 years) and the prevailing view of parents’ rights to make decisions on behalf of their adolescent children [14]. Implementation was restrained further by public and professional perceptions of young people’s rights and abilities to take responsibility for decisions affecting their health.

This paper builds on our insights into the acceptability of the new consent procedures published elsewhere [14]. Here, we consider the extent to which young women were able to exercise autonomy within the context of the HPV vaccination programme. We consider the perspectives of young women, parents and professionals, and how this was influenced by the content and form of the information provided.

## Methods

In this study, we define young people (and young women) as those aged between 10 and 24 years according to the World Health Organisation definition [15].

### Recruitment and consent to participate

The University of Bristol’s Faculty of Health Sciences Research Ethics Committee and the National Health Service (NHS) Health Research Authority provided the required approvals (references: 57621 & 18/HRA/0367, respectively).

The research was undertaken in two local authorities in the south-west of England where uptake rates of the HPV vaccination programme were ranked below the national average and implementation of the new consent procedures took place [12]. School recruitment and data collection occurred 18 and 2018/18 and 2018/19 programme years. Mainstream schools in which at least 12 female Year 8 students were not vaccinated during the 2016/17 programme year were sent information about the study and invited to participate. Of the 15 schools identified, four (26.7%) consented to take part. All alternative education provider settings (*n* = 17) were invited to participate in the study, of which five (29.4%) consented. Observations of the vaccination sessions (*n* = 3) took place in three of the mainstream schools during which detailed field notes recorded the context and any specific incidents relevant to uptake.

Topic guides were developed to cover the same key issues (beliefs about the HPV vaccine, views and experiences of the HPV vaccination programme, and opinions about the new consent procedures) with some adaptations relevant to the differing roles of immunisation nurses, mainstream school staff, alternative education providers, parents and young women (see Supplementary Material 1).

Semi-structured digitally recorded interviews were undertaken with 53 participants. This included interviews with the immunisation programme manager and three immunisation nurses who represent the entire permanent team delivering the HPV vaccination programme in the two local authorities. Our experience of undertaking research about the HPV vaccination programme shows that, in most schools, there is one key member of staff who has responsibility for delivery and organisation of the HPV vaccination programme. Depending on the school, this may be a member of the administration team, or a school staff member with additional responsibilities for Year 8 students (e.g. Head of Year). We undertook interviews with three key members of staff from the four mainstream schools that participated in the study (one school staff member at one of the mainstream schools was unable to participate in the study timescales). A key member of staff from each of the five alternative provider settings recruited to the study were interviewed.

Overall, 19 young women participated. Young women (*n* = 8) aged 12 to 13 years who were involved in the new consent procedures were recruited and interviewed at their school. Young women (*n* = 11) aged 13 to 17 years were recruited from community organisations. Of the 19 young women interviewed: all attended mainstream schools and had received the HPV vaccine, eight were from minority ethnic groups; 12 returned a signed parental consent form (one of whom had signed the form herself), six received the vaccine following parental verbal consent at the vaccination session, and one self-consented.

Finally 22 parents (21 mothers and one father) were recruited through community organisations since attempts to recruit parents through the school setting proved unsuccessful. Five parents had daughters who took part in the study. Depending on the recruitment procedure in each community organisation and individual preferences, young women and parents were interviewed separately, with their parent/daughter or with a peer/peers. Individual interviews were conducted with school staff and members of the immunisation team. All interviews were undertaken by one researcher (HF) and took place within community organisations, schools, homes or workplaces as preferred by the participant.

All interviewees aged 16 years or older gave written informed consent before participating in the study. For participants aged younger than 16 years, both parental consent and young women’s assent were required prior to participation.

All recordings were transcribed verbatim and thematic analysis [16] was undertaken assisted by NVivo 12 software. We used both an inductive and deductive approach to analyse the content, focusing on our main research questions while identifying key issues emerging from the data. Coding of all transcripts was undertaken by one researcher (HF), while a second researcher (MF) double-coded a sub-set of 12 transcripts to check for meaning, relevance and reliability, and to agree the coding framework applied to the full set of transcripts. Sections of text relating to young people’s autonomy within the HPV vaccination programme and communication of information were extracted. Themes were identified within which similarities and differences were explored.

## Results

The results presented provide a summary of the key issues relating to young women’s autonomy during the HPV vaccination programme. Illustrative quotations were chosen because they express concisely and typify responses relating to the themes.

### Extent of young people’s autonomy

#### Schools-based vaccination sessions

Young people’s independent and autonomous participation within the HPV vaccination programme is influenced by the environment (usually the school setting) in which it is delivered. Young people’s movements and behaviours are controlled and regulated in schools, by the timetable, curriculum, and policies enforced by school staff.

Researcher observations during vaccination sessions highlighted a clear dichotomy between the young women and adults (school staff and immunisation nurses) present. To maintain an orderly system, school staff frequently used their authority to control behaviours where young women were excitable and vocally expressed worries about receiving the vaccine. These anxieties related to the size of the needle and anticipated pain from receiving the HPV vaccine. Despite often protesting verbally, young women were mostly co-operative with instructions given by adults during the vaccination session. Where parental consent, either through paper-based consent forms or verbally, had been obtained, it was rare for young women to exercise autonomy and refuse the vaccination during the session.

Despite the legal framework and local policy supporting adolescent self-consent, the immunisation team appeared more comfortable in retaining parents as the responsible party for consent provision. This was reflected in their interactions with students: ‘*The trouble is, I need to speak to them [the parents]*’ [immunisation nurse, fieldwork, mainstream school 1]; ‘*Yes, you’re quite right, she [student’s mother] does want you to have the vaccine*’ [immunisation nurse, fieldwork, mainstream school 10]. Young women were complicit with this and appeared to prefer or expect their parents to be responsible for providing consent. This resulted in young women frequently deferring power to their parents, or other adults, to influence whether they received the vaccine or not: ‘*If my mum picks up [the phone], I’m having the jab*’ [student, field work, mainstream school 1].

The constraints routinely applied against young people exercising choice in a school setting may lead some young women to avoid vaccination by simply not attending school on the day of session. During interviews, some parents mentioned their daughters were absent for vaccination sessions. A few adult participants suggested it may be intentional, but the extent to which this happens remains unclear:

‘*I bet a lot of children don’t even turn up [to school], if they know it’s [the vaccination session] happening on Friday*’ [Mother 1, community group 2]

‘*You’re always going to have a very small number, thankfully, that will stay off school on that day to avoid having the injection regardless of what you do to promote the importance of it and the benefits of it*.’ [School staff 1, school 2]

#### Exercising autonomy during the consent procedures

The routine procedure of initially seeking written parental consent promotes the primacy of parental consent in the HPV vaccination programme. Within this, young women play an important role in ensuring the ‘success’ of the procedures, considered as the receipt of a completed parental consent form by the immunisation team. The young woman is expected to act as a ‘vehicle to consent’ by promptly delivering the consent form to her parents or carers, ensuring they record their wishes on the form, and finally returning the consent form to the appropriate member of staff at the school ahead of the vaccination session. One parent considered this an imbalance of power and responsibility inherent within the consent procedures:

‘*Even though it’s prioritising parental consent, you’re putting that responsibility on the child to get that important literature home and get it processed and get it back into school but they’re not actually responsible for it. It’s kind of quite strange*.’ [Mother 6, community group 6]

Interview participants frequently commented how this process granted opportunities for young women to exercise power through intercepting consent procedures. This could be achieved by young women simply not presenting the paper-based consent form to their parents:

‘*I think that’s why my daughter wasn’t like ‘mum can you sign this?’ [HPV vaccine consent form], you know, ‘cos she didn’t want it*.’ [Mother 9, community group 1]

Other strategies included not returning the completed parental consent form to the school, or even filling in the consent form themselves:

‘*They think if they hide the form, they don’t need to have it [the vaccine] and it’s amazing how many forms miraculously appear out of the bags when you say that you’re going to phone the parents*.’ [Immunisation nurse 3]

‘*Sometimes they don’t want to get it [the HPV vaccine] done so they forge the form.*’ [Young woman 1, parent verbal consent, mainstream school 10]

Participants felt this behaviour would be played out if there were worries about receiving the injection, rather than strongly formed beliefs opposed to vaccinations:

‘*If they’re scared the needle’s going to be really big they just won’t give it [the consent form] to their parents.*’ [Daughter 2, parent written consent, community group 5]

‘*If she [her daughter] knows it’s for an injection, she’ll probably throw it in the bin or something ‘cos that’s what she’s like. I mean that’s what most girls are like isn’t it? If they don’t want to have- well who wants to have an injection?*’ [Mother 4, community group 1]

To overcome barriers to the receipt of consent forms, one school augmented the primacy of parental consent by posting forms directly to parents. Students at this school were all in agreement that this approach was warranted for the reasons discussed above. These students considered if they were to be given the responsibility to deliver the consent forms to their parents, they would still prefer the school ensured their parents were aware that they should anticipate the arrival of a consent form:

‘*I think people [school staff] should get- like, if they wanted to give us the consent form, they should send home a text or ring my mum*.’ [Young woman 3, parent verbal consent, mainstream school 9]

Lack of priority towards receiving the HPV vaccine meant that young women could unintentionally intercept the consent process, because they forgot about or misplaced the parental consent form. This barrier to uptake could be overcome, in part, by seeking verbal consent from parents on the day of the vaccination session:

‘*I always want to give my mum the letters but I have a bad habit of putting things in my bag and then forgetting about it*.’ [Young woman 5, parent verbal consent, mainstream school 9]

‘*I know I had it in school but I came in the morning and I lost it ‘cos I was going to hand it in to reception but I sat down in this area and I lost it.*’ [Young woman 1, parent verbal consent, mainstream school 10]

School staff and immunisation nurses suggested that a student’s background could influence the extent to which additional efforts were required to ensure compliance with consent form receipt:

‘*It’s often though, the case that students who come from a more kind of disorganised background are the ones that don’t bring their forms back in. I know it’s an obvious thing to say but those that are out of routine, those are the ones where forms stay in bags or get left on the kitchen table or accidentally picked up, put in the bin and you won’t get them returned and those are the ones you’re chasing a lot*.’ [School staff 1, mainstream school 2]

Despite the constraints of the school environment, and consent procedures where adults hold greater influence, in exceptional circumstances young women could use their power to ensure they received the HPV vaccine. This could be through signing on behalf of their parents, or not returning completed paperwork where their parent had refused consent:

‘*We got given like a big sheet and my mum didn’t want me to get that [the HPV vaccine] or the meningitis I think, so I signed them myself and got it done anyway*.’ [Young woman 1, written consent, community group 4]

‘*So one in particular the young girl had come in, spoken to my colleague, gone down ringing mum, no reply, does mum want you to have the vaccine? Yes, yes, she wants me to, we just haven’t bought the form back ... Great girl, went through everything, really competent, signed her consent, someone else, another member of staff went on and gave the vaccine ... By the time we got back to the office she’d obviously rung mum then and mum had rung in absolutely fuming that she had signed, I believe she had signed as a refuser and the form hadn’t made its way to us.*’ [Immunisation nurse 2]

The structure of the consent procedures, with the expectation that the parent completes a consent form, could undermine young women’s autonomy despite their willingness and advocacy to receive the HPV vaccine:

‘*My mum kept forgetting. I kept reminding her but she kept forgetting to give it [the consent form] back to me.*” [Young woman, adolescent self-consent, mainstream school 1]

### Communication channels about the HPV vaccination programme

#### Information provision for young women

Information leaflets about the HPV vaccination programme, together with forms requesting parental consent, are routinely distributed by the school to parents or carers, either by the young woman taking the information home or by posting it to the home address. Perceptions of adults as the decision-makers and targets for information, undermined opportunities for young women to be informed about the HPV vaccine and involved in decisions affecting their health:

‘*It wasn’t targeted at us I don’t think. They [the school] just kind of gave us the letter and like oh you’re getting it in a few weeks.*’ [Young woman 4, parent written consent, community group 6]

Participants suggested that provision of information leaflets alone would be insufficient to engage young women about the HPV vaccine:

‘*I’m pretty sure most people probably didn’t read the leaflet, they probably just gave it to their parents.’* [Young woman 2, parent written consent, community group 4]

‘*You need to guide them through it a bit more rather than just sending information and expecting them to read it and act on it. I think they probably wouldn’t at a young age*.’ [Mother 1, community group 5]

Among families, different levels of communication and opportunities to engage young women about the HPV vaccination programme were evident. Presentation of the consent form to parents could act as a prompt for dialogue about the HPV vaccine and an opportunity to address young women’s information needs:

‘*I had to give her [mother] the consent form to sign the consent form for my vaccine and then we just talked about what the vaccine was for and then why boys don’t get it*.’ [Young woman 3, parent written consent, community group 6]

In other cases, there were limited opportunities to discuss the HPV vaccine within families. Seeking parental verbal consent during the vaccination session could further remove an opportunity for young women to find out about the HPV vaccine:

‘*My mum didn’t really tell me anything about it [the HPV vaccine]. Just the person [immunisation nurse] spoke to her on the phone what it was about and then just said it’s fine.’* [Young woman 3, parental verbal consent, school 9]

A few participants indicated cultural or religious preferences, promoting sexual relations solely in the context of marriage, could also inhibit communication within some families. This may also be influenced by parents’ perceptions of appropriateness of discussing this information with their vaccine-eligible daughters (12 to 13 years):

‘*Some ethnicities and cultures are, how shall I say it, slightly more hesitant shall we say about having injections and the reasons for it and the discussion of illness and disease and other more what might be considered sensitive matters like sex education for example, is either considered a taboo or can just be a really awkward matter that just isn’t discussed at home.*’ [School staff 1, mainstream school 2]

‘*It’s quite a tricky age to have those sorts of conversations [about sexual transmissibility of HPV], isn’t it? I guess it’s probably why it’s better if it’s just done- if it’s just rolled out, they just don’t really have a choice. I guess they do have to have a choice don’t they? That’s the problem.*’ [Mother 7, community group 1]

Levels of communication about the HPV vaccine with young women also varied within the school setting. Some young women recalled receiving information in assemblies or tutor time. More frequently, information leaflets were relied on as the primary method to communicate and involve young women in the HPV vaccination programme:

‘*It was like kind of unexpected, like we didn’t have assembly or we weren’t really told by any teachers, we were just told oh you’re getting a vaccine done and that was it and then it was like oh if you have any questions there will be a paper which will be given out which will tell you all the information you need. And then the day came and then we didn’t really like have anyone to question*.’ [Young woman 5, parent verbal consent, school 9]

In one case, an administrative oversight resulted in a group of vaccine eligible young women, who attended an alternative education provider co-located within a mainstream school, not being invited to receive the HPV vaccine with their peers:

‘*That’s communication failure then because we’ve missed that … Ours [vaccine eligible students] have not had the letters so that’s worth- Yeah, I’ll chase it up.*’ [School staff 1, alternative education setting 4]

#### Young women’s communication preferences about the HPV vaccine

Almost all study participants were supportive of increasing provision of age appropriate information for young women about the HPV vaccine. Schools were widely considered an acceptable setting, where educational sessions could be delivered through assemblies or Personal, Social, Health Education (PHSE) lessons:

‘*I think maybe probably like an assembly, or just like talking to the children about it I think would be better.*’ [Young woman 3, parent written consent, community group 6]

Face-to-face methods of communication were favoured, which could be supplemented with videos. There were mixed opinions as to who would be most appropriate to deliver educational sessions. External providers, such as healthcare professionals, may in some cases be preferable to school staff:

‘*I would think more a healthcare professional because people wouldn’t want to listen to teachers to be completely honest. When the teacher starts talking at you, it’s when people generally switch off, but at least if it’s someone external they try to listen*.’ [Young woman 1, parent written consent, community group 4]

Young women valued information explaining the risk and benefits of the HPV vaccination. Practicalities about what to expect on the day of the vaccination session were also frequently mentioned. This included information such as where the vaccination session was taking place in the school, how many doses were required, and whether the size of the needle increased for the second injection. This was felt beneficial with the potential for improving young women’s experience of having the HPV vaccine in the school setting, and mitigating misinformation and the circulation of rumours:

‘*When no one tells you, the girls just start, well the girls at my school just started making stuff up. Oh, the needles are really long and you’re going to die and stupid stuff like that and that got some of girls really scared so it’s good to give them at least some information so they know the basics*.’ [Young woman 1, parent written consent, community group 5]

It could also provide young women with an opportunity to provide informed consent, especially critical when parental consent forms were unreturned:

‘*I think if you have sessions within schools then that’s a lot more structured, you have to focus, you have to learn … so that’s something that has to happen, but if it’s a leaflet that can get lost or screwed up, that’s got so much potential to not get anywhere and then you get to the day and the kids like yeah I want the vaccine, you’re like great, your parents haven’t done this, you don’t know what it’s for, like what are we meant to do?’* [Young woman 2, parent written consent, community group 6]

#### Information provision for parents

As young women may wish to discuss vaccination with their families, parental information needs could also influence young women’s levels of understanding about the HPV vaccine. Where parental beliefs were underpinned by a favourable understanding of the biomedical model for vaccination, the provision of information leaflets may be sufficient for parents to consent for their daughter to receive the HPV vaccine:

‘*We got a leaflet just saying this was the vaccine that she was going to have, very kind of basic information about what HPV is, I think. Other than that, yeah, that was it, just for me to consent and of course we did*.’ [Mother 1, community group 3]

However, the content of the leaflets was not always accessible to parents:

‘*If they could just put the information out in clearer form everybody would be able to understand it.*’ [Mother 1, community group 1]

Based on limited understanding of the information leaflets, some parents appeared to lack confidence in deciding whether to provide consent for their daughter to be vaccinated. Healthcare professionals were viewed as trustworthy sources of information and could successfully provide assurance for positive vaccine decision-making:

‘*I just remember when she [her daughter] came in with that form [for the HPV vaccine] from the school nurse, it was in a specialist setting … you just call the school nurse … and ask all the questions and she just reassured me about all of it so I knew it was ok to do it. All I can remember is that it was about cervical cancer, I can’t remember what all the rest was about.*’ [Mother 2, community group 2]

There were mixed opinions as to whether that information could be effectively delivered to parents within in the school setting:

‘*I would say do a talk on it but then you might not get many parents turn up*’. [Mother 9, community group 1]

Parents also sought further information or clarification about the HPV vaccine through the internet. This was almost always accompanied by a recognition that the legitimacy of the information could be compromised:

‘*Then you google and then you see the scare story, and then you don’t want to have it [the HPV vaccine] done*.’ [Mother 3, community group 2]

‘*When you search something on the internet obviously there needs to be some way that the parent can distinguish between the two because there’s always going to be one for and one against and they’re both going to be telling it from their point of view, and yes they’re both possibly correct. But they’re both probably wrong to a certain- in some way*.’ [Father 1, community group 1]

The availability of misinformation about vaccines was also discussed by the immunisation team, whilst retaining parents as being responsible for understanding the information and making the decision about their daughter’s vaccination:

‘*If they’re [parents] reading information that isn’t right, it’s coming from an anti-vacc- It does read quite legitimately but we know as practitioners that what it’s saying is incorrect information. As a layperson you wouldn’t necessarily know that so as long as we’re putting out the right information as well so they can make that informed decision that is their right to do that.*’ [Health manager, immunisation team]

Due to language and literacy issues, school staff recognised that reliance on information leaflets as the sole communication channel presented a barrier to some parents being able to provide informed consent. Additional support would be required to overcome barriers to understanding:

‘*If there are parents who have their own learning needs, we would probably need to be talking to them, not just sending the note home.*’ [School staff 1, alternative education setting 1]

‘*There’s still a few parents here who can’t read so hopefully the students would explain to them*.’ [School staff 1, mainstream school 1]

## Discussion

The findings from this qualitative study suggest young women’s autonomy within the HPV vaccination programme is currently constrained by the school structure and consent procedures, both of which favour adult authority over young people’s rights to participation. Some young women and their parents appear to have difficulties in engaging with information leaflets - the predominant method used to inform families about the HPV vaccination programme - which further undermines young women’s participation in decision-making and consent procedures. This may ultimately influence vaccination uptake.

Considerable academic debate remains as to how young people’s rights should be exercised [17], and the extent of implementation in practice in the healthcare setting more generally [18].

### Improving communication about the HPV vaccine

Schools facilitate efficient access to the majority of vaccine-eligible young people and are a widely acceptable setting for delivery of vaccination programmes [19]. However, this study demonstrates challenges in communicating vaccine messages in schools-based vaccination programmes where paper-based leaflets are used as the primary source of information for families. This contributes further evidence to a previous study which identified unmet information needs about the HPV vaccine among families [20]. Further, information leaflets rely on parental literacy which has been shown to exclude around 15% of parents from receiving accessible information [21]. Provision of information through leaflets limited opportunities for the immunisation team to interact face-to-face with families, or to frame and target specific HPV vaccine messages to families with additional information needs.

Delivery of educational sessions to address young people’s information needs in the school setting were widely acceptable. In response to young people’s unmet information needs, and recommendations developed from this qualitative study, we are undertaking a study to co-produce an educational package jointly with young people for delivery in schools with lower uptake [22]. The educational package will aim to increase uptake of the HPV vaccine, by addressing young people’s information needs, and increasing their autonomy in consent procedures.

There is increasing recognition that vaccine hesitancy, delays in accepting or refusing vaccines despite the availability of vaccination services, contributes to lower uptake [23]. Lower vaccine confidence, the trust in the effectiveness and safety of vaccines, in addition to the healthcare system that delivers them, may also contribute to inequity [24]. Improving communication of evidence-based vaccine messages, and responding to misinformation circulating in social media and anti-vaccination activities, have been proposed as strategies to address vaccine hesitancy and improve vaccine confidence [25–27].

Research related to this study has shown parents have information needs about vaccines which could be addressed. For example, written reasons for refusal included concerns related to safety and side-effects, insufficient research evidence, and parental choice. Some parents thought that the vaccine was not needed, or they preferred to delay vaccination for their daughters. The qualitative findings from this study support that healthcare professionals are a trusted source of information and can support parents in positive decision-making. Effective communication strategies delivered by healthcare professionals could change how vaccine hesitant families think and feel about the HPV vaccine, leading to higher and more equitable uptake.

### Interventions to improve communication about the HPV vaccine

Some other approaches to improving communication with families show promise. In 2017, Danish health authorities launched a media campaign, which included a series of educational videos, to increase health literacy and restore public confidence in response to negative media reports related to the safety of the HPV vaccine [28]. The campaign was associated with restoration of uptake of their HPV vaccination programme to its baseline level [28]. Recent evidence from a randomised controlled trial undertaken in a healthcare setting suggested uptake could be increased through motivational interviewing by healthcare professionals and supplementary information for HPV vaccine-hesitant parents [29, 30]. However, in the context of the English, schools-based HPV vaccination programme there is currently a lack of evidence-based interventions to address parental information needs.

#### Strengths and limitations

This study has, from the perspectives of different stakeholders and young women from a range of backgrounds, examined the extent to which young people are autonomous in the English schools-based HPV vaccination programme. We did not seek the perspectives of young men as part of this study as data collection was undertaken when the English HPV vaccination programme was offered to young women only. Although not intentional, recruitment of adult participants was weighted towards females with just three male participants (one father and two male school staff). We did not collect socio-demographic information from all participants to establish ethnicity or social class. Therefore, the extent to which the findings from this study are relevant to young men and fathers, or differ by ethnicity or social class, is unknown.

## Conclusions

Young women’s active participation and independence within the HPV vaccination programme is constrained by the setting of vaccination, the primacy of parental consent procedures, and unmet information needs among families. Strategies to improve communication, and address families’ information needs, could increase young people’s autonomy in consent procedures and result in higher uptake of the HPV vaccination programme.

## Supplementary Information

**Additional file 1.**

## Data Availability

The datasets generated and analysed during the current study are not publicly available as consent from study participants was not obtained for the use of the data in this way. Annoymised datasets may be made to available to other researchers with permission from the Principal Investigator of the study on reasonable request.

## Abbreviations

HPV: Human papillomavirus
PSHE: Personal, Social, Health Education
