# Supplementary material for: Young women’s autonomy and information needs in the schools-based HPV vaccination programme: a qualitative study
Source: BMC Public Health. 2020 Nov 10;20:1680. doi: 10.1186/s12889-020-09815-x (PMC7654043; doi:10.1186/s12889-020-09815-x)
Supplement: Supplementary file 1 — Additional file 1. [file 12889_2020_9815_MOESM1_ESM.docx]

**Young Women’s Topic Guide**

[Note: *Ensure that the participants have read the information sheet and signed the consent form before the discussion starts*.]

I’d like to hear about your views and experiences about consent for the HPV vaccine. This is very informal; you can talk about anything you think is important for us to know. Remember, your answers to our questions will not be considered “right” or “wrong”, because I want to know about what you think.

**HPV vaccine**

Before you were offered the HPV vaccine, had you ever heard of HPV?

Can you tell me about what you know about HPV now?

How did you find out about the HPV vaccine?

What information were you given?

What do you think are the positive things about the HPV vaccine are?

What do you think the negative things about the HPV vaccine are?

**Information for parents**

Do you know what information was given to your parents or carers about the HPV vaccination programme in school?

Did your parents or carers discuss it with you? If yes, what did they say? Prompt: Did they think it was a good or a bad idea for you to have the vaccination? Did they think the school was a good or a bad place to have the vaccine?

Were there any disagreements about the HPV vaccine? E.g. one of you thinking it was a good idea to have the vaccine and another person thinking it wasn’t? If yes, how was the disagreement sorted out?

**Consent**

What do you understand by the word consent?

- *If participant not sure, explain that consent is giving permission for something to happen*

What do you think are important things to consider when taking consent? Prompt: Information needs?

Who do you think can give permission for you to have the HPV vaccine? Prompt: Parent? Teacher? Healthcare professional? Yourself?

How do you think you can give permission to have the HPV vaccine? Prompt: Speaking? Written down? Not saying no?

Do you think it’s important to ask the permission of:

- Young people. Prompt: Age?
- Parents/carers

What do you think should happen if -

- The parent says that they do not want their Year 8 (12-13 years old) daughter to have the vaccine, but she wants to have it? Prompt: Should the student be asked anything? Should her parents be contacted?
- The parent says that they do not want their Year 10 (15-16 years old) daughter to have the vaccine, but she wants to have it? Prompt: Should the student be asked anything? Should her parents be contacted?
- The parent/carer form has not been returned and a young person wants the vaccine? Prompt: Phone call to parent? Young person’s consent?
- The parent/carer wants the daughter to have the vaccine, but the daughter refuses? Prompt: Should the student be asked anything? Should her parents be contacted?

The school gave out a form to ask parents and carers if they agreed that their daughter should have the HPV vaccination. Did you see that form? Did your parent/carer fill out the form?

Were you or your parent/carer reminded to bring the consent form back to the school? If yes, who reminded you and/or your parent/carer?

Our records show that your parent/carer form was not returned to the school. Do you know why?

Do you think sending out a form to parents/carers is a good way of getting permission to have the HPV vaccine? Can you think of any other ways? Prompt: Email? Telephone? Opt-out? Give consent once for all vaccines? Young people?

Who decided whether you should have/not have the vaccine?

Who helped with the decision? Prompt: Mother? Father? Other family members? Friends? Teacher? School nurse?

Did you realise that there was a new process this year to allow young women to give permission to have the vaccine? Do you think that is a good idea?

Do you think you had enough information to be able to give permission to have the vaccine?

Do you think that young women should be able to give permission to have the HPV vaccination in school without the consent of their parents/carers?

**Receiving the HPV vaccine**

Did you receive the HPV vaccine? If yes, how did you feel?

Did you have any concerns or worries? If yes, what were these?

**After the vaccination programme**

Do you think you made the right decision about whether or not to have the vaccine? Why do you feel that way?

Did you talk with your parents/carers about it afterwards? If yes, what did they say?

Did your parents contact the school after you had the vaccine? If yes, what did they say?

Do you have any suggestions about how to improve the method of giving permission to have the HPV vaccination? If yes, what?

**Finally, is there anything else you would like to tell me or ask me about?**

**Many thanks for taking part in this interview.**

[NOTE: *ensure that each participant is given the £10 gift voucher*]

**Parent/Carer Topic Guide**

[Note: *Ensure that the participant has read the information sheet and signed the consent form before the interview starts*.]

I’d like to hear about your views about the HPV vaccine. This is very informal; you can talk about anything you think is important for us to know. Remember, your answers to our questions will not be considered “right” or “wrong”, because I want to know about what you think.

**Vaccinations**

Can you tell me a little about what you think about vaccinations in general?

Did your daughter have her childhood vaccinations? Why? **HPV vaccine**

Before your daughter was offered the HPV vaccine, had you ever heard of HPV?

Can you tell me about what you know now about HPV/HPV vaccine?

What do you think the positive things about the HPV vaccine are?

What do you think the negative things about the HPV vaccine are?

**Information for parents**

What information did you receive about the HPV vaccination programme in your daughter’s school?

Did you discuss it with your daughter? If yes, what did you say? Prompt: Did you think it was a good/bad idea for her to have the vaccination?

Do you think the school is a good/bad place to have the vaccine? Why?

Were there any disagreements about the HPV vaccine? E.g. one of you thinking it was a good idea to have the vaccine and another thinking it wasn’t? If yes, how was the disagreement sorted out?

**Consent**

What do you understand by the word consent?

What do you think are important things to consider when taking consent? Prompt: Information needs?

Who do you think can give consent for your daughter to have the HPV vaccine? Prompt: Your daughter? Teacher? Healthcare professional?

How do you think you consent can be provided for the HPV vaccine? Prompt: Speaking? Written down? Complying with healthcare professionals?

Do you think it’s important to ask consent from:

- Young people. Prompt: Age?
- Parents/carers

**Consent Case Studies**

*Case 1*

The parent says that they do not want their Year 8 daughter (12-13 years old) daughter to have the vaccine, but she wants to have it?

- *What do you think should happen?*
- *What do you think the student should be asked?*
- *Should the parents be contacted?*

*Case 2*

The parent says that they do not want their Year 10 (15-16 years old) daughter to have the vaccine, but she wants to have it?

- *What do you think should happen?*
- *What do you think the student should be asked?*
- *Should the parents be contacted?*

*Case 3*

The parent/carer form has not been returned and a young person wants the vaccine?

- *What do you think should happen?*
- *What do you think the student should be asked?*
- *Should the parents be contacted?*

*Case 4*

The parent/carer wants the daughter to have the vaccine, but the daughter refuses?

*- Anything the student could be asked?*

*- Should her parents be contacted?*

*Case 5*

A Year 8 student (aged 12-13) presents for the HPV vaccination with a form that has not been completed properly – the ‘I agree’ and ‘I do not agree’ boxes have both been ticked.

- *What do you think should happen?*
- *What do you think the student should be asked?*
- *Should the parents be contacted?*

**The parental consent form**

Did you receive a form from the school asking you whether or not you wanted your daughter to have the HPV vaccination?

Did you complete the form?

Did you receive any reminders to send the form back to the school? If yes, how? Prompt: email, telephone, through your daughter?

Our records show that your parent/carer form was not returned to the school. Do you know why?

Do you think sending out a form to parents/carers is a good way of getting consent for the HPV vaccine? Can you think of any other ways? Prompt: Email? Text? Opt-out? Consent once for all vaccines? Young people consent?

Who gave consent for whether your daughter should have/not have the vaccine? Prompt: Mother? Father? Daughter?

Can you tell me a little bit about how this decision was made?

Was there any disagreement about the decision e.g. daughter agrees but parent does not? If yes, how was this resolved?

Did anyone else help with the decision? Prompt: Other family members? Friends? Teacher? School nurse?

Were you aware that there was a new process this year to allow young women to consent for themselves to have the vaccine? Do you think that is a good idea?

Do you think your daughter had enough information to be able to consent to have the vaccine?

Do you think that young women should be able to consent to have the HPV vaccination without the permission of their parents/carers?

**Receiving the vaccine**

Did your daughter receive the HPV vaccine?

**After the vaccination programme**

Do you think the right decision was made about whether or not your daughter should have the vaccine? Why do you feel that way?

Do you have any suggestions for improvements to the HPV vaccination consent process? If yes, what?

**Finally, is there anything else you would like to tell me or ask me about?**

**Many thanks for taking part in this interview.**

[NOTE: *ensure that each participant is given the £10 gift voucher*]

**School Staff Topic Guide**

[Note: *Ensure that the participant has read the information sheet and signed the consent form before the interview starts*.]

**Background questions**

What is your current job title and role in the school?

How long have you worked at this school?

**HPV vaccine**

What do you know about the HPV vaccine?

What is your opinion about delivering the HPV vaccination in schools?

Prompt: Is this a school responsibility? Positive and negative aspects of delivering the vaccine in a school setting.

Can you explain to me your role in the HPV vaccination programme?

How are Year 8 female students informed out about the HPV vaccination programme?

How are their parents/carers informed about the HPV vaccination programme?

**Consent**

What do you understand by the word consent?

What do you think are important things to consider when taking consent? Prompt: Information needs?

Who do you think can give consent for Year 8 students to have the HPV vaccine? Prompt: The student? Teacher? Healthcare professional? Parents?

How do you think consent can be provided for the HPV vaccine? Prompt: Speaking? Written down? Complying with healthcare professional?

Do you think it’s important to ask consent from:

- Young people. Prompt: Age?
- Parents/carers

**Consent Case Studies**

Case 1

A Year 10 student (aged 15-16) presents for HPV vaccination without a signed consent form from her parents but would like to be vaccinated.

*What do you think should happen?*

*What do you think the student should be asked?*

*Should the parents be contacted?*

Case 2

A Year 8 student (aged 12-13) wants to receive the HPV vaccine but her parents do not agree.

*What do you think should happen?*

*Do you think the student should be assessed for competency to consent for herself?*

*Should the parents be contacted?*

Case 3

A Year 10 student (aged 15-16) presents for vaccination with a signed consent form from her parents but states that she does not want to be vaccinated.

*What do you think should happen?*

*What do you think the student should be asked?*

*Should the parents be contacted?*

Case 4

A Year 8 student (aged 12-13) presents for the HPV vaccination with a form that has not been completed properly – the ‘I agree’ and ‘I do not agree’ boxes have both been ticked..

*What do you think should happen?*

*What do you think the student should be asked?*

*Should the parents be contacted?*

**School consent procedures**

What are the school procedures for returning parental consent forms for the HPV vaccine?

Do parents receive reminders to return the consent form?

If yes, how?

Do female students receive reminders to return the consent form?

If yes, how?

Approximately how many female students do not return the parental consent form?

What do you think are the main reasons why the parental consent form is not returned?

Do you think parents should always provide consent as to whether their daughter has the vaccine?

Do you think young women should be able to provide consent about whether to have the vaccine?

Are you aware of any disagreements about the decision e.g. daughter agrees but parent does not? If yes, how was this resolved?

Do you think sending out a form to parents/carers is a good way of getting permission to have the HPV vaccine? Can you think of any other ways? Prompt: Email? Text? Telephone? Opt-out? Give consent once for all vaccines? Young people?

**Experience of the implementation of the new consent procedures**

Were you aware of the new consent procedures taking place for the HPV vaccination programme in your school?

Could you tell me a little bit about your understanding of the new self-consent procedures?

- How was it organised?
- Who was involved and what was their role? E.g. School staff, school nurses, support staff?

Thinking about the Year 8 students:

- How were the young women told about the self-consent procedures?
- What information were they given?
- Do you think they were sufficiently informed?
- Where did it take place?
- How did the young women respond to the self-consent procedures?
- Overall, did they think it was a good thing or not a good thing?
- Were there any obvious differences of opinion between the Year 8 students? If yes, what factors do you think influenced these differences of opinion?

Now thinking about the parents/carers:

Before the vaccination session in the school:

- How were the parents/carers told about the self-consent procedures?
- What information were they given?
- Do you think they were sufficiently informed?
- Did any parents/carers return the form to say they did not want their daughter to receive the HPV vaccine (parental opt-out)? If yes, did they give a reason?
- Did any of the parents/carers contact the school about girls being able to give self-consent for the vaccine? If yes, what did they say?

After the vaccination session in the school:

- Did any parents contact the school after their daughter self-consented for vaccination? If yes, what did they say?
- Were there any obvious differences of opinion between the parents? Prompt: Some parents not happy about the new procedures, some people thinking the nee procedures are better. If yes, what factors do you think influenced these differences of opinion?

Thinking about this first year of the new consent process:

- Overall, what do you think has worked well?
- Overall, what do you think hasn’t work so well?

Do you have any suggestions for improvements to the HPV vaccination consent process? If yes, what?

**Finally, is there anything else you would like to tell me or ask me about?**

**Many thanks for taking part in this interview.**

[NOTE: *ensure that the participant is given the £10 gift voucher*]

**Healthcare Professional Topic Guide**

[Note: *Ensure that the participant has read the information sheet and signed the consent form before the interview starts*.]

**Background questions**

How long have you been an immunisation nurse?

What is your current job title and role?

How long have you been delivering the HPV vaccination programme?

**HPV vaccine**

What do you know about the HPV vaccine?

What do you think the positive things about the HPV vaccine are?

What do you think the negative things about the HPV vaccine are?

Can you explain to me your role in the delivery of the HPV vaccination programme?

What is your opinion about delivering the HPV vaccination in schools?

Prompt: Positive and negative aspects of delivering the vaccine in a school setting. Does this vary between schools?

How are Year 8 female students informed out about the HPV vaccination programme?

How are their parents/carers informed about the HPV vaccination programme?

**Consent Case studies**

Case 1

A Year 10 student (aged 15-16) presents for HPV vaccination without a signed consent form from her parents but would like to be vaccinated.

*What would you do?*

*What would you ask the student?*

*Would you contact the parents?*

Case 2

A Year 8 student (aged 12-13) wants to receive the HPV vaccine but her parents do not agree.

*What would you do?*

*Would you assess if the student is competent to consent for herself?*

*Would you contact the parents?*

Case 3

A Year 10 student (aged 15-16) presents for vaccination with a signed consent form from her parents but states that she does not want to be vaccinated.

*What do you think should happen?*

*What do you think the student should be asked?*

*Should the parents be contacted?*

Case 4

A Year 8 student (aged 12-13) presents for the HPV vaccination with a form that has not been completed properly – the ‘I agree’ and ‘I do not agree’ boxes have both been ticked.

*What do you think should happen?*

*What do you think the student should be asked?*

*Should the parents be contacted?*

**Consent procedures**

Can you tell me a little bit about the procedures for obtaining parental consent?

Prompt: who takes responsibility for this, the school or the school nurses?

Do parents receive reminders to return the consent form?

If yes, how?

Do female students receive reminders to return the consent form?

If yes, how?

Approximately how many female students do not return the parental consent form?

What do you think are the main reasons why the parental consent form is not returned?

Do you think parents should provide consent as to whether their daughter has the vaccine?

Do you think young women should be able to consent about whether to have the vaccine or not?

Are you aware of any disagreements about the decision e.g. daughter agrees but parent does not? If yes, how was this resolved?

Do you think sending out a form to parents/carers is a good way of getting permission to have the HPV vaccine? Can you think of any other ways? Prompt: Email? Text? Telephone? Opt-out? Give consent once for all vaccines? Young people?

**Experience of the implementation of the self-consent procedures**

Could you tell me a little bit about your experiences of the new consent procedures?

- How was it organised?
- Who was involved and what was their role? E.g. School staff, school nurses, support staff?

Thinking about the Year 8 students:

- How were they told about the self-consent procedures?
- What information were they given?
- Do you think they were sufficiently informed?
- Where did it take place?
- How did the young women respond to the self-consent procedures?
- Overall, did they think it was a good thing or not a good thing?
- Were there any obvious differences of opinion between the Year 8 students? If yes, what factors do you think influenced these differences of opinion?

Now thinking about the parents/carers?

- How were the parents/carers told about the self-consent procedures?
- What information were they given?
- Do you think they were sufficiently informed?
- Do you know if any of the parents/carers returned the form to say they did not want their daughter to receive the HPV vaccine (parental opt-out)? If yes, do you know the reason why they did not want their daughter(s) to receive the vaccine?
- Do you know if any of the parents/carers had any questions or concerns about the self-consent procedures? If yes, what did they say?
- Are you aware of any differences of opinion between the parents? If yes, what factors do you think influenced these differences of opinion?
- Did any parents contact the school after their daughter self-consented for vaccination? If yes, what did they say?

Thinking about this first year of the new consent process:

- Overall, what do you think has worked well?
- Overall, what do you think hasn’t work so well?
- Do you have any suggestions for improvements to the HPV vaccination consent process? If yes, what?

**Finally, is there anything else you would like to tell me or ask me about?**

**Many thanks for taking part in this interview.**

[NOTE: *ensure that the participant is given the £10 gift voucher*]
